# Contrasting effects of Deadend1 (*Dnd1*) gain and loss of function mutations on allelic inheritance, testicular cancer, and intestinal polyposis

**DOI:** 10.1186/1471-2156-14-54

**Published:** 2013-06-17

**Authors:** Jennifer L Zechel, Stephanie K Doerner, Angela Lager, Paul J Tesar, Jason D Heaney, Joseph H Nadeau

**Affiliations:** 1 Department of Genetics and Genome Sciences, Case Western Reserve University School of Medicine, Cleveland OH 44106, USA; 2 Department of Molecular and Human Genetics, Baylor College of Medicine, Houston, TX 77030, USA; 3 Pacific Northwest Research Institute, 720 Broadway, Seattle, WA 98122, USA

**Keywords:** Testicular cancer, Allelic segregation, Intestinal neoplasia, DND1

## Abstract

**Background:**

Certain mutations in the Deadend1 (*Dnd1*) gene are the most potent modifiers of testicular germ cell tumor (TGCT) susceptibility in mice and rats. In the 129 family of mice, the *Dnd1*^*Ter*^ mutation significantly increases occurrence of TGCT-affected males. To test the hypothesis that he *Dnd1*^*Ter*^ allele is a loss-of-function mutation; we characterized the consequences of a genetically-engineered loss-of-function mutation in mice, and compared these results with those for *Dnd1*^*Ter*^.

**Results:**

We found that intercrossing *Dnd1*^*+/KO*^ heterozygotes to generate a complete loss-of-function led to absence of *Dnd1*^*KO/KO*^ homozygotes and significantly reduced numbers of *Dnd1*^*+/KO*^ heterozygotes. Further crosses showed that *Dnd1*^*Ter*^ partially rescues loss of *Dnd1*^*KO*^ mice. We also found that loss of a single copy of *Dnd1* in *Dnd1*^*KO/+*^ heterozygotes did not affect baseline occurrence of TGCT-affected males and that *Dnd1*^*Ter*^ increased TGCT risk regardless whether the alternative allele was loss-of-function (*Dnd1*^*KO*^) or wild-type (*Dnd1*^*+*^). Finally, we found that the action of *Dnd1*^*Ter*^ was not limited to testicular cancer, but also significantly increased polyp number and burden in the *Apc*^*+/Min*^ model of intestinal polyposis.

**Conclusion:**

These results show that *Dnd1* is essential for normal allelic inheritance and that *Dnd1*^*Ter*^ has a novel combination of functions that significantly increase risk for both testicular and intestinal cancer.

## Background

Testicular germ cell tumors (TGCTs) are the most common cancer affecting young men. They are disproportionately represented in men aged 20–40, comprising ~60% of all cancers in this age group [1], while accounting for only 1.0-1.5 % of all cancers [2]. The incidence of TGCTs has increased in the last 30 years [3], rising approximately 3% per year from 1972 to 2002 [4] compared to an overall annual decrease of 0.6% from 1994–2009 for all cancers in men [5].

Testicular cancer is widely considered to be one of the most heritable forms of cancer [6,7]. Genetic factors contribute significantly to TGCT susceptibility as exemplified by a 4- to 15-fold increased risk in the sons and brothers of affected men, respectively [8,9]. With conventional inheritance however, risk should be similar in sons and brothers. The ~4-fold difference in occurrence of affected sons and brothers implies that other modes of inheritance are involved. Despite strong heritability, known genetic mutations and single nucleotide polymorphisms (SNPs) make only modest contributions to susceptibility, illustrating the genetically complex nature of this disease. The *gr/gr* deletion, which is a common cause of infertility in men, is associated with increased TGCT risk [10]. But its contribution is modest, occurring in only 3% of TGCT cases with a family history and 1% of unaffected individuals [11]. *gr/gr* results from a 1.6 Mb deletion at the *AZFc* locus at Yq11 of the human Y chromosome, a region that contains multiple copies of several genes that are involved in male germ cell development including *DAZ* (deleted in azospermia), *BPY2* (basic charge, Y-linked 2) and *CDY1* (chromodomain protein, *Y*-linked 1). Effects of the g*r/gr* deletion on germ cell development and differentiation are largely unknown.

Several single nucleotide polymorphisms (SNPs) are associated with increased TGCT risk in humans. In particular, six studies implicate SNPs that are associated with at least 14 genes: *ATF7IP, BAK1, DMRT1, KITLG, SPRY4, TERT, HPGDS, MAD1L1, RFWD3, TEX14, RAD51C, PPM1E, DAZL* and *PRDM14*[12-19]. Molecular mechanisms remain unclear, in part because many SNPs are located outside the coding region of the associated gene, and in part because the haplotype structure and sequence of these loci have not yet been fully reported. Interestingly, the *TERT* SNP is also associated with adenocarcinoma [20].

**Figure 1:**
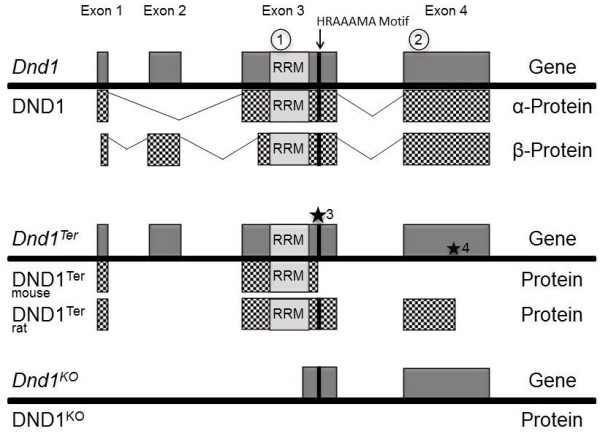
**Structure of the *Dnd1*, *Dnd1***^***Ter***^**and *Dnd1***^***KO ***^**genes and their inferred predicted protein products.** Gene arrangements are displayed above the solid line, and the corresponding protein product is displayed below the solid line. *Dnd1* has two isoforms: *α-Dnd1* and *β-Dnd1* which differ in the amino-terminus of the protein. *Dnd1* has an RNA recognition motif (RRM) in the C-terminal portion of exon 3. The HRAAAMA motif that is presumably part of the putative ATPase domain is located between amino acids 181–186 in the mouse genome. SNPs identified in human TGCTs include a (1) Glu86Ala [25] and (2) an Asp219Glu [24]. In the mouse, the *Ter* mutation creates a premature stop-codon at amino acid 178 (★3), which is located 37 nucleotides from the 3′ most exon-exon junction. The rat *Ter* mutation has the premature stop codon at amino acid 289 (★4) within exon 4. The RRM is intact in both mouse and rat *Dnd1* mutants allowing these proteins to possibly recognize and bind to target RNAs; the putative ATPase domain is lost in the mouse *Dnd1*^*Ter*^ and truncated in rat *Dnd1*^*Ter*^. The *Dnd1*^*KO*^ allele retains the 3′ most portion of exon 3 and exon 4, but does not have a transcriptional start site.

Certain mutations in the *Dead End 1* (*Dnd1*) gene are potent modifiers of TGCT susceptibility in both mice and rats. In the mouse, the spontaneous *Dnd1*^*Ter*^ mutation significantly increases TGCT susceptibility in the 129 family of inbred mouse strains. In particular, *Dnd1*^*Ter*^ increases occurrence of TGCT-affected males from a baseline of ~5% in the 129S1/SvImJ strain to 17% in *Dnd1*^*+/Ter*^ heterozygotes and 94% in *Dnd1*^*Ter/Ter*^ homozygotes [21,22]. The *Ter* mutation is a single base substitution in exon 3, 3′ to its single RNA recognition motif (RRM), that transforms an arginine residue to a premature stop codon (Figure 1, see also [22]). The *Ter* mutation has been proposed to produce an mRNA that is lost by nonsense-mediated degradation owing to the presence of the premature stop codon. This conclusion was based on northern blots of mRNA isolated from TGCTs in *Dnd1*^*Ter/Ter*^ males [22]. But the tissue type (TGCTs) used in this study may not have been appropriate to assess the fate of *Dnd1* transcripts. 

In the rat, a spontaneous mutation has been identified where a G to A substitution in exon 4 produces a premature stop codon that is thought to result in a 62 amino acid truncation at the C-terminus of the DND1 protein (Figure 1, see also [23]). This mutation leads to germ cell tumors in males and females as well as to spontaneous metastases.

 In humans, sequencing of TGCT candidate genes in several large studies failed to detect a significant number of *DND1* mutations [4,24]. Of the two SNPs that were identified, one (Glu86Ala) is located within the conserved RRM of DND1 (Glu86Ala). The functional consequences of this mutation on *Dnd1* expression and function or on TGCT risk are not known.

*Dnd1* has many unique functions. *Dnd1* shares significant sequence similarity with *A1cf*, a gene that encodes the RNA binding subunit of the Apobec1 cytidine deaminase that edits specific sites in specific mRNAs [22]. Interestingly, DND1 blocks access of specific miRNAs to their 3′ target in mRNAs such as *p27*, *LATS2* and *TDRD7*[26]. DND1 also binds several pluripotency factor mRNAs including *Oct4*, *Sox2*, *Nanog* and LIN28 [27], regulators of cell cycle including *LATS2*, *TP53*, *p21* and *p27*[27,28], apoptotic factors such as BCLX and BAX [27], and is a positive regulator of geminin mRNA translation through binding to its 3′ UTR [29]. The role of DND1 in these functions and complexes is unknown. DND1 transports mRNA transcripts from germ cell nuclei to germ cell granules [30]. Finally, *Dnd1* is required for primordial germ cell (PGC) survival [31]; PGCs are the stem cell for TGCTs [32]. Together these observations implicate *Dnd1* in many aspects of RNA translation control.

The *Dnd1* gene in mouse has four exons and encodes an α-isoform, which is 352 amino acids, and a β-isoform, which is 340 amino acids (Figure 1). These isoforms are derived through alternative splicing and differ by 12 amino acids at the amino-terminus of the protein. DND1-α is expressed in early embryos, whereas DND1-β is expressed in the germ cells of the adult testis [33]. Both isoforms contain a single RNA recognition motif (RRM) and a highly conserved HRAAAMA motif (unpubl). This 7 amino acid motif is found in most orthologues of *Dnd1* and *A1cf* (JHN, unpubl.). In zebrafish, ATPase activity has been attributed to a variant of this motif in *Dnd1*[34].

Several considerations suggest that the assumption that *Dnd1*^*Ter*^ leads to loss-of-function is erroneous. *In vivo* loss of function using morpholinos to reduce expression in zebrafish showed that *Dnd1* is required for PGC survival [31], possibly through an ATPase activity [34]. Although TGCTs have been reported in zebrafish [35-37], reduced *Dnd1* expression was not sufficient for tumorigenesis. However, because genetic background strongly regulates TGCT susceptibility in mice and rats [23,38-40], absence of TGCTs in the zebrafish study could implicate either the nature of the mutation or genetic background as critical elements determining tumorigenic outcome.

To test whether TGCT susceptibility depends on the nature of *Dnd1* mutations, we generated a line of 129/SvImJ-*Dnd*^*KO*^ mice. We found that deficiency of *Dnd1* leads to highly biased allelic inheritance and that *Dnd1*^*Ter*^ partially restores normal inheritance. We also found that *Dnd1*^*Ter*^ acts distinctly from *Dnd1*^*KO*^ to increase TGCT risk in a dosage-dependent manner in 129S1/SvImJ males, whereas *Dnd1*^*KO*^ did not significantly affect susceptibility. Finally, recent work suggests that susceptibility to both TGCTs and intestinal polyposis share genetic predisposition [41] and that DND1 is normally expressed in the intestine [42-46]. We therefore tested whether *Dnd1*^*Ter*^ affects intestinal tumorigenesis and found that *Dnd1*^*Ter*^ significantly increases polyp number and burden in *Apc*^*+/Min*^ mice.

## Results

### Loss of *Dnd1* homozygotes and heterozygotes

Unexpected results were found for *Dnd1*^*+/KO*^ intercrosses that were intended to produce *Dnd1*^*+/KO*^ heterozygotes and *Dnd1*^*KO/KO*^ homozygotes as well as *Dnd1*^*+/+*^ wild-type controls for TGCT surveys. The genotypic distribution among adult mice differed significantly from 1:2:1 Mendelian expectations (Table 1; χ^2^ = 108.4, p<0.0001; two-tailed test, 2 degrees of freedom). In particular, no *Dnd1*^*KO/KO*^ homozygotes were observed among 282 offspring (Table 1). To assess the extent of the deviation from expectations, we assumed that the number of *Dnd1*^*+/+*^ mice (N = 120) corresponded to expectations, and then extrapolated from this wild-type number to the expected numbers of *Dnd1*^*+/KO*^ heterozygotes (N = 240) and *Dnd1*^*KO/KO*^ homozygotes (N = 120) mice. In addition to a complete deficiency of *Dnd1*^*KO/KO*^ homozygotes, only 68% (= 162/240) of the expected number of *Dnd1*^*+/KO*^ heterozygotes was found, suggesting that parental *Dnd1*^*+/KO*^ heterozygosity either biased segregation against the *Dnd1*^*KO*^ allele, or alternatively that partial or complete loss of *Dnd1* function led to lethality for *Dnd1*^*KO/KO*^ homozygotes and reduced viability for *Dnd1*^*+/KO*^ heterozygotes.

**Table 1:** ***Dnd1***^***+/KO ***^**intercross – genotype distribution at weaning**

**Genotype**	**Observed**	**Mendelian expectations**
*Dnd1*^*+/+*^	120	70.5
*Dnd1*^*+/KO*^	162	141
*Dnd1*^*KO/KO*^	0	70.5

*Dnd1*^*KO*^ segregation in intercrosses and backcrosses. Results are presented after pooling data for both genders (Tables 1, 2, 3, 4), because occurrence of females and males did not differ significantly (not shown).

To examine the developmental timing of genotypic loss, we time-mated heterozygous *Dnd1*^*+/KO*^ males and females, and flushed embryonic day 3.5 (E3.5) embryos from the oviduct. Embryos (N=25) were individually cultured for one week before genotyping. The genotypic distribution differed significantly from 1:2:1 Mendelian expectations (Table 2; χ^2^ = 22.4, p<0.0001; two-tailed test, 2 degrees of freedom). No *Dnd1*^*KO/KO*^ homozygotes were found among 25 cultured embryos, the number of *Dnd1*^*+/KO*^ heterozygotes was significantly reduced, and no evidence was found for unfertilized oocytes. In addition, the genotypic distributions for E3.5 embryos (Table 2) and for adult mice (Table 1) did not differ significantly (not shown). These results confirm observations found among adult mice and suggests that reduced *Dnd1* function either biased segregation, or led to early embryonic lethality. Interestingly, unusual inheritance patterns have also been reported for *Apobec1*^*KO*^ – *Dnd1*^*Ter*^ interactions tests [47] and for Apobec1 complementation factor *A1cf*, the paralog of *Dnd1*[48].

**Table 2:** ***Dnd1***^***+/KO ***^**intercross – genotype distribution at embryonic day 3.5 (E3.5)**

**Genotype**	**Observed**	**Mendelian expectations**
*Dnd1*^*+/+*^	16	6.25
*Dnd1*^*+/KO*^	9	12.5
*Dnd1*^*KO/KO*^	0	6.25

*Dnd1*^*KO*^ segregation in intercrosses and backcrosses. Results are presented after pooling data for both genders (Tables 1, 2, 3, 4), because occurrence of females and males did not differ significantly (not shown).

To test whether allelic segregation in *Dnd1*^*+/KO*^ is inherently biased, we examined the genotypic representation in reciprocal backcrosses of *Dnd1*^*+/KO*^ heterozygotes to *Dnd1*^*+/+*^wild-type mice. Segregation did not differ significantly from 1:1 Mendelian expectations for either maternal or paternal heterozygosity (Table 3). Interestingly, occurrence of *Dnd1*^*+/KO*^ heterozygous progeny differed in intercrosses versus backcrosses of *Dnd1*^*+/KO*^ heterozygous parents, where a significant deficiency of *Dnd1*^*+/KO*^ heterozygotes was found among intercross but not backcross progeny (Table 3). These results suggest that segregation in each parent is normal and that gametes in each parent are comparably functional in backcrosses but not intercrosses.

**Table 3:** ***Dnd1***^***+/KO ***^**segregation in backcrosses versus intercrosses**

**Cross**	**No. *Dnd1***^***+/+***^	**No. *Dnd1***^+/KO^*(expectations)	**% expected number**	**Litter size**
Intercross	120	162 (240)	67.5	5.6±0.5 n=18
Backcross:maternal	110	107 (110)	97.2	5.8±0.4 n=30
Backcross:paternal	100	96 (100)	96	5.2±0.4 n=19

*Dnd1*^*KO*^ segregation in intercrosses and backcrosses. Results are presented after pooling data for both genders (Tables 1, 2, 3, 4), because occurrence of females and males did not differ significantly (not shown).

*Mendelian expectations based on *Dnd1*^*+/+*^; litter size (mean ± SEM, *n* is the number of litters).

Litter size can be used to determine whether unusual inheritance patterns result from embryonic lethality. Litter size should be reduced substantially if deficiency of some genotypes results from lethality. By contrast, if production of gametes or fertilization is biased towards particular alleles, litter sizes should not differ among the various crosses. However, despite significant deviation from the expected genotypic distributions (Table 1), litter sizes did not vary significantly among crosses (Table 3). These results argue against lethality as the cause of the unusual genotypic distributions.

Based on the premise that the DND1^Ter^ protein is translated instead of being lost through nonsense mediated degradation, a single copy of *Dnd1*^*Ter*^ may be sufficient to at least partially rescue *Dnd1*^*KO/KO*^ loss. To test this hypothesis, we intercrossed mice heterozygous for the *Dnd1*^*KO*^ and *Dnd1*^*Ter*^ mutations and examined occurrence of the four genotypic classes among the resulting offspring (Table 4). The distribution of *Dnd1*^*+/+*^*, Dnd1*^*+/KO*^*, Dnd1*^*+/*Ter^*, Dnd1*^*KO/Ter*^ offspring differed significantly from 1:1:1:1 Mendelian expectations (*χ*^*2*^ = 9.1, p<0.03; two-tailed test, 3 degrees of freedom). The genotype with the largest deviation from expectations was the *Dnd1*^*KO/Ter*^ heterozygote, which supports the hypothesis that the DND1^Ter^ protein is translated and retains sufficient functionality to enable at least partial viability of animals in the absence of wild-type DND1; if *Dnd1*^*Ter*^ conferred loss of function, then loss of both *Dnd1*^*KO/KO*^ and *Dnd1*^*KO/Ter*^ should have been found.

**Table 4:** **Interaction between *Dnd1***^***Ter ***^**and *Dnd1***^***KO***^

**Genotype**	**Observed**	**Mendelian expectations**
*Dnd1*^*+/+*^	40	32.5
*Dnd1*^*+/KO*^	41	32.5
*Dnd1*^*+/Ter*^	29	32.5
*Dnd1*^*KO/Ter*^	20	32.5

*Dnd1*^*KO*^ segregation in intercrosses and backcrosses. Results are presented after pooling data for both genders (Tables 1, 2, 3, 4), because occurrence of females and males did not differ significantly (not shown).

Male progeny (N=130) from crosses between *Dnd1*^*+/Ter*^ and *Dnd1*^*+/KO*^ mice.

### *Dnd1* mRNA levels in *Dnd1*^*+/KO*^ males

**Figure 2:**
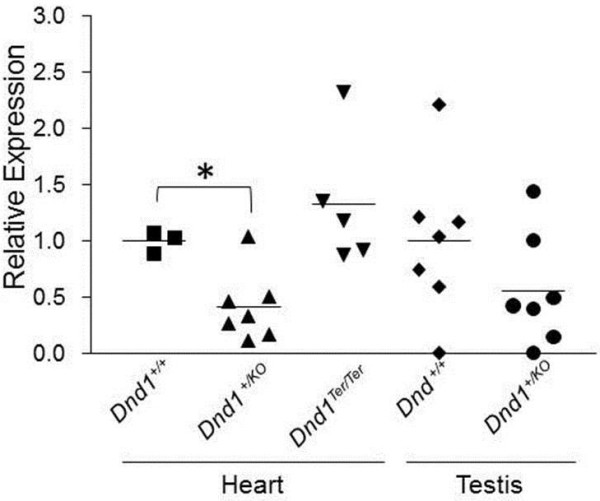
***Dnd1 *RNA levels in heart and testes.***Dnd1* expression cannot be measured reliably in *Dnd1*^*Ter/Ter*^ testes because they deficient in germ cells.

To determine whether the *Dnd1*^*KO*^ allele leads to reduction of *Dnd1* mRNA, *Dnd1* transcript levels were measured in heart and testis from *Dnd1*^*+/KO*^ and wild-type littermate control males that were 6–8 weeks old. *Dnd1*^*+/KO*^ mRNA levels were significantly reduced in heart (0.41 ± 0.12; t = 3.1, p<0.05; two-tailed t-test, n=7) and substantially reduced in testis (0.56 ± 0.19; n=7) compared to their wild-type littermate controls (1.0 ± 0.06 and 1.0 ± 0.26; n=3 and 7, respectively) (Figure 2). *Dnd1* mRNA levels from *Dnd1*^*Ter/Ter*^ hearts (1.33 ± 0.26) did not differ significantly from wild-type controls. Testis samples were not tested because severe germ cell deficiency in *Dnd1*^*Ter/Ter*^ males would compromise interpretation of any results [21,22,36]. These results confirm that *Dnd1*^*KO*^ but not *Dnd1*^*Ter*^ mice showed reduced *Dnd1* mRNA levels.

### Germ cell numbers and fecundity in *Dnd1*-deficient males

We next sought to test whether *Dnd1*^*Ter*^ and *Dnd1*^*KO*^ alleles induce similar effects on PGC numbers in testes of heterozygous and wild-type littermates from the *Dnd1*^*KO*^ line. Testes from *Dnd1*^*+/Ter*^ males showed modest but significant reductions in germ cell numbers and testis weight [22,49]. We weighed testes from 6–10 week old *Dnd1*^*+/KO*^ males and their wild-type (+/+) littermates to determine if they were phenotypically similar to those from *Dnd1*^*+/Ter*^. Testes from wild-type males weighed an average of 4.43 ± 0.88g, which was similar to the average testes weight of 4.44 ± 0.69g in *Dnd1*^*+/KO*^ heterozygotes (Additional file 1: Figure S1A). These results suggest that germ cell numbers were not significantly reduced in *Dnd1*^*+/KO*^ males. Histological analysis confirmed these results for both *Dnd1*^*+/Ter*^ and *Dnd1*^*+/KO*^ males (Additional file 1: Figure S1B & C). Finally, both male and female heterozygotes for the *Dnd1*^*KO*^ allele were fertile as measured by the similar number of offspring produced in backcrosses to 129S1/SvImJ (Table 3). Together these results suggest that the *Dnd1*^*KO*^ allele did not negatively impact germ cell numbers or fertility.

### *Dnd1* mutations and TGCT-affected males

To test whether partial *Dnd1* deficiency affects occurrence of affected males, *Dnd1*^*+/KO*^ and their *Dnd1*^*+/+*^ wild-type control littermates derived from *Dnd1*^*+/KO*^ intercrosses were surveyed for spontaneous TGCTs (Table 5). Significant differences were not detected for occurrence of TGCT-affected *Dnd1*^*+/KO*^ heterozygous and *Dnd1*^*+/+*^ wild-type males. By contrast, 39% of *Dnd1*^*+/Ter*^ males are affected, which is significantly higher than the occurrence of affected *Dnd1*^*+/KO*^ and their *Dnd1*^*+/+*^ males (p<0.0001; Fisher’s exact test, two-tailed, 1 degree of freedom). (This rate for *Dnd1*^*+/Ter*^ males in our colony is significantly higher than published reports [50]. The rate has been consistently higher for several years, without obvious explanation. Because of the study design, this increased rate does not affect results or interpretation in the present work.) Thus, the *Dnd1*^*Ter*^ but not the *Dnd1*^*KO*^ mutation increases TGCT risk.

**Table 5:** ***Dnd1 *genotype and occurrence of males affected with at least one spontaneous TGCT**

**Genotype**	**Affected**	**Unaffected**	**Affected males (%)**
*Dnd1*^*+/+*^	8	121	*6%
*Dnd1*^*+/KO*^	7	109	*6%
ǂ*Dnd1*^*+/Ter*^	35	90	39%

*Dnd1*^*+/KO*^ males are offspring of both *Dnd1*^*+/+*^ × *Dnd1*^*+/KO*^ and *Dnd1*^*+/KO*^ × *Dnd1*^*+/+*^ reciprocal crosses. ǂ Results from Zechel *et al.*[51].

To test whether the increased occurrence of affected *Dnd1*^*Ter/Ter*^ males was a result of increased *Dnd1*^*+/Ter*^ or the absence of *Dnd1*^*+/KO*^, we crossed *Dnd1*^*+/Ter*^ and *Dnd1*^*+/KO*^ animals to generate compound heterozygous *Dnd1*^*Ter/KO*^ offspring that were surveyed for TGCTs (Table 6). A single copy of *Dnd1*^*Ter*^ increased occurrence of TGCT-affected males from a baseline of 15% for *Dnd1*^*+/+*^ wild-type males to 39% for *Dnd1*^*+/Ter*^ males. A second copy of *Dnd1*^*Ter*^ (*Dnd1*^*Ter/Ter*^) further increased occurrence of affected males to 98% [21,22]. We then asked whether the higher occurrence of affected *Dnd1*^*Ter/Ter*^ males was due to the absence of wild-type DND1, or a result of an increased dosage of *Dnd1*^*Ter*^. Compound heterozygous males (*Dnd1*^*KO/Ter*^) have a rate that does not recapitulate the rate found for *Dnd1*^*Ter/Ter*^ homozygotes (35% versus 97.8%, respectively) (Table 6). Instead, the rate for compound heterozygotes was similar to the rate (34% and 39%) *Dnd1*^*+/Ter*^ heterozygote. These results suggest that DND1^Ter^ protein has dose-dependent effects on TGCT risk.

**Table 6:** **Occurrence of TGCTs-affected males of various *Dnd1 *genotypes**

**Genotype**	**Affected**	**Unaffected**	**Affected males (%) (%)**
ǂ*Dnd1*^*+/+*^	6	34	15%
ǂ*Dnd1*^*+/KO*^	1	28	3%
ǂ*Dnd1*^*+/Ter*^	14	27	34%
ǂ*Dnd1*^*KO/Ter*^	7	13	35%
*Dnd1^*+/Ter*^	35	55	ǂ39%
*Dnd1^*Ter/Ter*^	45	1	ǂ98%

*Dnd1*^*+/KO*^ and *Dnd1*^*+/Ter*^ animals were intercrossed to test for occurrence of males with at least one TGCT. ǂ Offspring from *Dnd1*^*+/KO*^ × *Dnd1*^*+/Ter*^ crosses; * offspring from *Dnd1*^*+/Ter*^ × *Dnd1*^*+/Ter*^. ǂData are from Zechel *et al.*[51].

### *Dnd1*^*Ter*^ and intestinal polyposis

**Figure 3:**
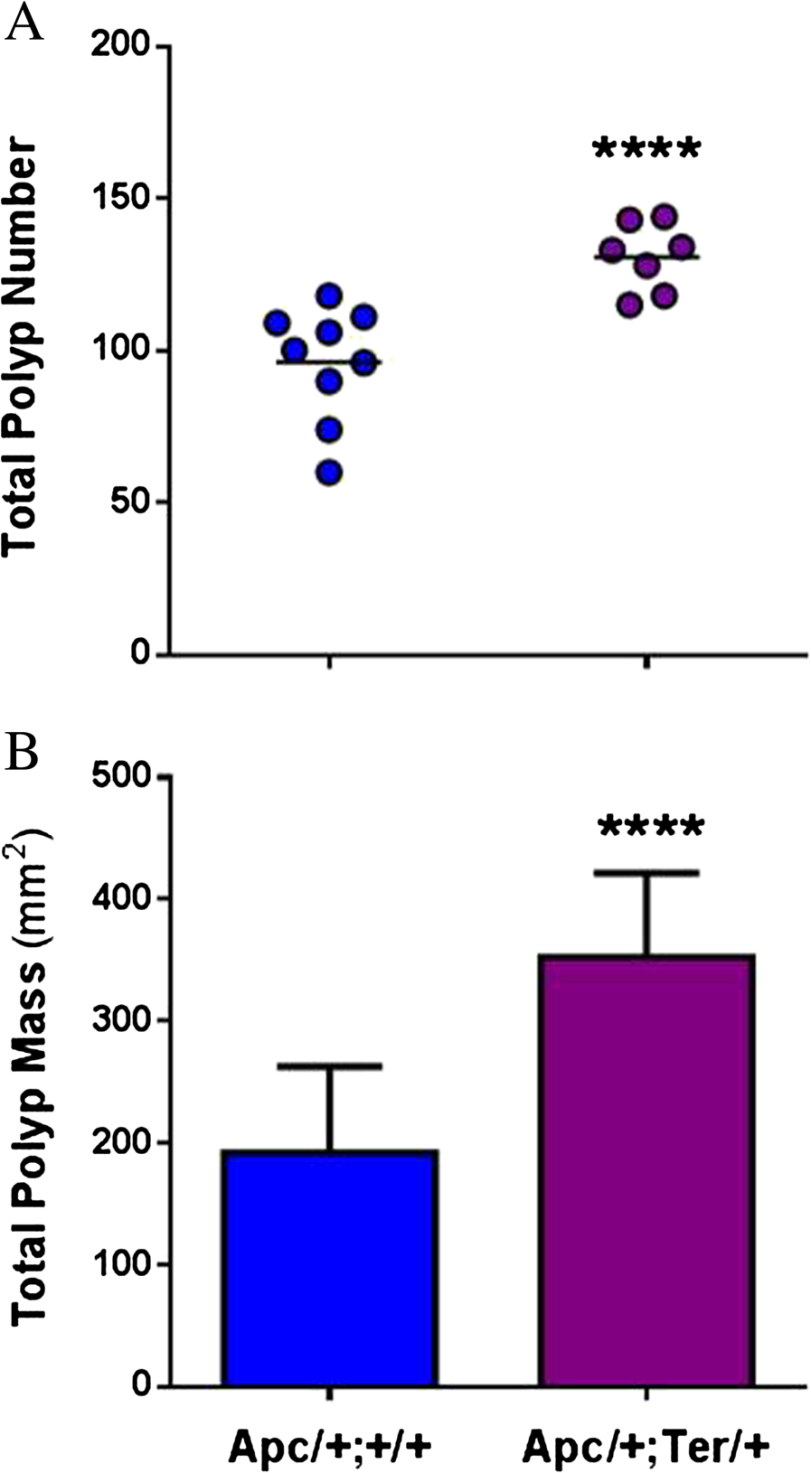
**Polyp number (A) and mass (B) in C57BL/6-*Apc***^***+/Min ***^***Dnd1***^**+/*Ter ***^**males.** Males were surveyed for polyp number and mass after 100 days on the 5010 diet.

Although *Dnd1*^*Ter*^ is a potent modifier of TGCT susceptibility, we speculated that the tumorigenic properties of *Dnd1*^*Ter*^ may also be relevant in the intestine where *Dnd1* is also expressed [52]. To test this hypothesis, we used the *Apc*^*+/Min*^ mouse model of intestinal polyposis. These mice develop numerous intestinal polyps at an early age and are a model of human Familial Adenomatous Polyposis (FAP) [53]. We crossed *Dnd1*^*+/Ter*^ and *Apc*^*+/Min*^ mice to generate compound *Apc*^*+/Min*^*Dnd1*^*+/Ter*^ heterozygotes (test) as well as a single-heterozygous *Apc*^*+/Min*^*Dnd1*^*+/+*^ control. After 100 days of age, a significant 1.5-fold increase in polyp number was observed in *Apc*^*+/Min*^*Dnd1*^*+/Ter*^ double-heterozygous test males compared to *Apc*^*+/Min*^*Dnd1*^*+/+*^ control males (130 ± 1.3 and 96.2 ± 1.4, respectively; t = 4.4, p<0.001, two-tailed t-test; Figure 3A). Similarly, total polyp mass was also significantly elevated from 192.2 ± 11.6 mm^2^ in *Apc*^*+/Min*^*Dnd1*^*+/+*^ mice to 352.6 ± 11.4 mm^2^ in *Apc*^*+/Min*^: *Dnd1*^*+/Ter*^ males (t = 4.5, p<0.0001; two-tailed t-test; Figure 3B), suggesting that a single copy of *Dnd1*^*Ter*^ exacerbates intestinal polyp initiation and development in mice that are genetically susceptible to intestinal polyposis and that the action of *Dnd1*^*Ter*^ is not limited to TGCTs.

## Discussion

TGCTs are the third most heritable form of cancer, with approximately 25% of the susceptibility being attributed to underlying genetic factors [7]. The limited success in identifying genetic variants that account for a significant proportion of TGCT cases highlights the complex nature of TGCT susceptibility [54-56]. Discovery of the *gr/gr* deletion, together with validated SNPs in the *ATF7IP, BAK1, DMRT1, KITLG, SPRY4, TERT, HPGDS, MAD1L1, RFWD3, TEX14, RAD51C, PPM1E, DAZL* and *PRDM14*[12-19,57]. Even with these findings however, the majority of inherited TGCT susceptibility remains unknown. In the mouse, several TGCT susceptibility genes have been identified, including *Kit*, *Kitlg*, *Pten*, *Dmrt1*, *Eif2s2* and others (for a review, see [58]). Together these human and mouse genes implicate pathways and functions related to germ cell proliferation, PGC differentiation and cell cycle control in TGCT development. But much remains to be learned about the nature of mutations in these genes that lead to tumorigenesis and about the ways that these molecular changes disrupt these pathways.

The current study suggests that *Dnd1*^*Ter*^ is not a loss-of-function allele, but instead affects TGCT risk and embryonic development in distinct ways from *Dnd1*^*KO*^, a true loss-of-function mutation. The location of the point mutation found within *Dnd1* does not conform to the “position of an exon-exon junction rule” for non-sense mediated decay [59]. The premature *Ter* stop codon is located 37 nucleotides upstream of the 3′-most exon-exon junction, within the 50–55 nucleotide range that fails to elicit nonsense-mediated decay (Figure 1). As a result, a translated DND1^Ter^ protein could be created consisting of *Dnd1* exons 1–2 and part of exon 3, with an intact RRM but a disrupted HRAAAMA. The RRM within DND1^Ter^ may still recognize its target mRNAs, but any functional or regulatory capabilities normally derived from the deleted portion of DND1 would be absent. This supposition is supported by work with the rat DND1^Ter^ mutation, where the GFP-DND1^Ter^ fusion protein is detectable in Huh7 cells [23]. The normal cellular processes that DND1^Ter^ might disrupt are numerous because RNA binding proteins have diverse functions, including alternative splicing, RNA stability, miRNA regulation, and translation control [60-63]. To date, antibodies are not available for mouse DND1, thereby precluding many obvious studies.

Recent work revealed an essential role of a putative ATPase domain in DND1 (RAAAE) in PGC survival [34]. This previously unannotated domain shares homology with RAAA (amino acids 178–181) in mouse DND1 where the arginine residue represents the amino acid substituted with a stop codon in *Dnd1*^*Ter*^[22]. Thus any ATPase activity in mouse DND1 possesses would be lost in DND1^Ter^. This region is also part of a larger motif in DND1, HRAAAMA, which is highly conserved and found in many paralogous RNA binding proteins, including Apobec complementation factor (A1CF), suggesting that this motif is functionally important in this gene family (unpubl.).

Although DND1^Ter^ appears to be a novel variant that increases TGCT risk, its tumorigenic effects are limited to the 129 strain of mice, which is not surprising, since this is the mouse strain that is susceptible to spontaneous TGCTs [38,63]. This finding highlights the importance of genetic background on TGCT susceptibility, which has been observed in humans, particularly in the disparate TGCT rates among ethnic groups [4,64]. The importance of genetic background is also evident in the interaction of DND1^Ter^ and mouse background. The 129 strain interacts positively with DND1^Ter^ to increase TGCT risk, whereas C56BL/6J and other strains only exhibit phenotypes related to some but not all aspects of mutations in genes such as *Kit*, *Kitl*, *Pten*, *Eif2s2I* and other TGCT modifier genes, but not TGCTs [55,65,66]. However, the tumorigenic effects of *Dnd1*^*Ter*^ are not limited to PGC transformation in the 129 strain mice. C57BL/6J mice with the *Apc*^*Min*^ mutation are highly susceptible to intestinal polyps [67]. However, when mice are compound heterozygotes for both *Apc*^*Min*^ and *Dnd1*^*Ter*^, polyp numbers and polyp burden are significantly increased (Figure 3). These results suggest that DND1^Ter^ affects pathways in both PGC transformation and intestinal polyposis.

Embryonic lethality is the usual interpretation for biased genotypic distributions in intercrosses, especially with complete loss of homozygous mutants. However this interpretation is difficult to reconcile with normal litter sizes. In particular, loss of ~50% of intercross progeny (Table 1) should lead to average litter sizes that are ~2 rather than ~4 pups per litter (Table 3). We are aware of very few examples where substantial embryonic lethality does not affect litter size [68]. Instead, we propose that the unusual genotypic distributions result from biased allelic inheritance where specific combinations of oocyte and sperm are favored (or disfavored). Normal segregation in reciprocal backcrosses with the 129S1/SvImJ suggests that gametes are produced in sufficient numbers and are adequately functional (Table 3). Instead results for intercrosses suggest that deficits in both females and males are needed to bias allelic inheritance. Together these results suggest that the bias arises at fertilization. Interestingly, genetically and functionally related genes also show biased segregation, including *Apobec1* and *A1cf*[47,48]

The contrasting effects of different classes of *Dnd1* mutations may explain why sequencing studies failed to yield mutations within *DND1* in human TGCT cases [24,25]. Complete loss of DND1 function is not sufficient to promote TGCT formation, even on the TGCT-susceptible 129S1/SvImJ inbred genetic background; reducing the chances that *DND1* mutations play a significant role in human TGCTs. Results from zebrafish support this interpretation. Loss of *Dnd1* inhibits PGC migration and results in PGC deficiency, but did not produce TGCTs [31]. TGCTs have been previously reported in zebrafish [35,49]. *Dnd1*^*Ter*^ homozygotes also show reduced migration and loss of PGCs, with typically less than 20 PGCs arriving to the presumptive fetal gonads [21]. Instead, our results show that it is the loss of the carboxy-terminus of DND1 protein that increases TGCT risk. The role of DND1^Ter^ in TGCT formation is further supported by recent results in a rat study where a spontaneous mutation producing a premature stop codon in exon 4, similar to *DND1*^*Ter*^, resulted in TGCTs [23]. To date, human *DND1* mutations have not yet been reported that yield a similarly truncated DND1 protein.

## Conclusions

Through intercrosses with mice carrying a complete loss of function *Dnd1*^*KO*^ allele our study showed that DND1 is necessary for embryonic viability and results in abnormal allelic segregation. We have further shown that *Dnd1*^*Ter*^, previously believed to be a loss-of-function allele, is likely translated into a protein that retains some normal DND1 function. Crosses with the *Dnd1* knockout and *Ter* alleles revealed that the effects of the *Ter* allele on TGCT incidence depend on *Ter* dosage. Expression of the *Ter* allele in an *APC*^*Min*^ model of intestinal polyposis also significantly increased polyp burden. These results demonstrate that *Dnd1*^*Ter*^ enhances tumorigenesis in two separate mouse models of cancer.

## Methods

### Mice

#### 129S1/SvImJ

This strain (JR002448, previously known as 129/SvImJ) was obtained from the Jackson Laboratory (Bar Harbor, ME, USA). All studies were conducted on this inbred genetic background.

#### 129S1/SvImJ-*Dnd1*^*+/KO*^

ES cells with a targeted deletion of *Dnd1* were generated from 129S6 mice by the Intrexon Corporation, (Blacksburg, VA). Exons 1–2 and most of exon 3 of *Dnd1* were removed through homologous recombination (Additional file 2: Figure S2), and cells were negatively selected with thymidine kinase (TK) and diphtheria toxin A (DTA), and positively selected with neomycin. PCR was used to confirm homologous recombination in these cells. ES cells were then injected into blastocysts (Case Transgenic and Targeting Facility) and the resulting chimeras were backcrossed onto the 129S1/SvImJ strain for more than 10 generations.

C57BL/6J (B6; JR000664) and C57BL/6J-*Apc*^*+/Min*^ (*Apc*^*+/Min*^; JR002020) mice were purchased from the Jackson Laboratory (Bar Harbor, ME).

From birth to 30 days of age, all mice were fed an autoclaved standard laboratory diet (Purina 5010 LabDiet (Richmond, IN) and were provided autoclaved water *ad libitum*. All mice were maintained on a 12-h light/dark cycle at the Wolstein Research Facility (CWRU).

Procedures were approved and conducted in compliance with Institutional Animal Care and Use Committee (IACUC) standards at (CWRU).

### Embryonic day 3.5 (E3.5) embryo cultures

Embryos from time-mated *Dnd1*^*+/KO*^ X *Dnd1*^*+/KO*^ pregnant females were flushed from the oviducts and uterine horns on embryonic day 3.5 (E3.5) and cultured as previously described [69]. To obtain sufficient material for reproducible genotyping, embryos were cultured for 1 week in individual wells of a 24-well tissue culture plate

### Genotyping

DNA for PCR genotyping was extracted from tail tissue (mice) or cell masses (E3.5 embryo cultures). The nucleotide substitution in the *Ter* mutation results in the creation of a *Dde1* site that was used for genotyping [22]. The *Dnd1*^*KO*^ allele was amplified using primers extending from exon 4 of *Dnd1* into the neomycin gene of the targeting construct. The *Dnd1*^*KO*^ primers are: CTGCGTGTTCGAATTCGCCAATGA (F), ACAAAGAGAAACCCGGTCTCGGAA (R). Primers used for genotyping *Apc*^*+/Min*^ were previously described [67].

### Tumor surveys

Male mice between the ages of 4–16 weeks were surveyed for TGCTs. Tumor incidence was calculated as the percentage of males with at least one TGCT. Histological analysis (H&E staining, see below) was used to confirm any TGCTs that were ambiguous at autopsy.

### Intestinal polyp survey

Mice were euthanized with cervical dislocation. The small and large intestines were immediately removed, flushed with cold PBS, and cut longitudinally for polyp measurements. Polyps were counted and cross-sectional diameter was measured in the small intestine and colon with a Leica MZ10F Modular Stereomicroscope. Individual polyp size and number were used to calculate a measure of total polyp mass for each mouse, and this measure was used as a surrogate for polyp burden.

### Histology

Testes were fixed with 10% formalin for at least 48h. Tissues were then embedded in paraffin and sectioned (5 μm) at the Case Comprehensive Cancer Center Tissue Procurement and Histology Core facility (TPHC). Hematoxylin and eosin staining was done in the TPHC facility.

### Quantitative real-time PCR

RNA was extracted from cells and tissues using the RNeasy micro- and mini-kits, respectively (Qiagen Inc., Valencia, CA) according to the manufacturer’s instructions and including an on-column DNAse treatment. RNA was reverse-transcribed using the qScript Synthesis Kit (Quanta BioSciences Inc., Gaithersburg, MD). Changes in relative expression were quantified with the Chromo4 real-time PCR system (MJ Research) and TaqMan primers to Dnd1(Invitrogen #Mm00849348) and normalized to 18S (Invitrogen #4319413E) using manufacturer protocols and reported as mean ± SEM.

### Statistical analysis

Data are presented as mean ± SEM. Two-tailed t-tests were used to evaluate results for quantitative PCR and data for both polyp numbers and burden. Chi-square contingency tests were used evaluate differences in occurrence of TGCT-affected males. Standard goodness-of-fit tests were used to evaluate differences between observed and Mendelian expectations for backcross and intercross segregation results. Then, to estimate the extent of loss for particular genotypes, the number of wild-type (+/+) mice (or embryos) was taken as the expected number for this genotypic class, and assuming Mendelian segregation (i.e. 1:2:1), the expected number of mutant heterozygotes and homozygotes was calculated. The difference between these observed and expected numbers was used to estimate the extent of loss for each genotypic class.

## Abbreviations

TGCT: Testicular germ cell tumor; SNP: Single nucleotide polymorphism; RRM: RNA recognition motif; PGC: Primordial germ cell; GFP: Green fluorescent protein.

## Competing interests

The authors declare that they have no competing interests.

## Authors’ contributions

JLZ and JHN conceived of the study, JLZ did the work with the exception of the data provided in Figure 3, which was conducted and analyzed by SKD. PJT did the embryo flushes and culturing. JDH assisted with initial transgenic mouse creation, experimental design and intellectual input. JHN assisted with the statistical analysis. JLZ and JHN wrote the original draft with editing done by AL, SKD and JDH. All authors read and approved of the final version.

## Supplementary Material

Additional file 1: Figure S1 Testis weight and histology in *Dnd1*^*+/+*^ and *Dnd1*^*+/KO*^. (A) Testes were removed and weighed from mice aged 6 to 10 weeks. Testes weights from adult heterozygous *Dnd1*-deficient males (Het, *Dnd1*^*+/KO*^) and their wild-type control littermates (WT, *Dnd1*^*+/+*^) were similar. Testes from wild-type males (WT) weighed an average of 4.43 ± 0.88g, compared to testes from heterozygous *Dnd1*^*+/KO*^ males (4.44 ± 0.69g). Body weights did not differ significantly between WT (*Dnd1*^*+/KO*^) and heterozygous (*Dnd1*^*+/KO*^) animals (data not shown). Histology of these testes show no morphological differences between *Dnd1*^*+/+*^ (B) and *Dnd1*^*+/KO*^ (C). These results suggest that testes in *Dnd1*^*+/KO*^ males do not show significant germ cell deficiency. Click here for file

Additional file 2: Figure S2 Generation of *Dnd1*^*KO*^ knockout mice. (A) Genomic *Dnd1* locus showing flanking *Hars* and *Wdr55* genes. Exons are shown as boxes and arrows show the direction of transcription. The left and right homology arms of the targeting construct are indicated. Following homologous recombination, exons 1, 2 and a portion of 3 of *Dnd1* are removed and a neomycin selection unit is introduced. The triangle next to the neomycin unit indicates direction of transcription. The *Hars* and *Wdr55* genes remain intact. Click here for file
