# Supplementary figures and images for: Contrasting effects of Deadend1 (Dnd1) gain and loss of function mutations on allelic inheritance, testicular cancer, and intestinal polyposis
Source: BMC Genet. 2013 Jun 17;14:54. doi: 10.1186/1471-2156-14-54 (PMC3693958; doi:10.1186/1471-2156-14-54)

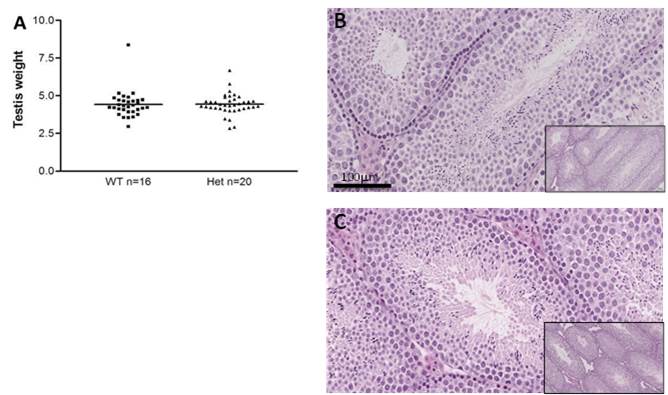

Supplement: Additional file 1: Figure S1 — Testis weight and histology in Dnd1+/+ and Dnd1+/KO. (A) Testes were removed and weighed from mice aged 6 to 10 weeks. Testes weights from adult heterozygous Dnd1-deficient males (Het, Dnd1+/KO) and their wild-type control littermates (WT, Dnd1+/+) were similar. Testes from wild-type males (WT) weighed an average of 4.43 ± 0.88g, compared to testes from heterozygous Dnd1+/KO males (4.44 ± 0.69g). Body weights did not differ significantly between WT (Dnd1+/KO) and heterozygous (Dnd1+/KO) animals (data not shown). Histology of these testes show no morphological differences between Dnd1+/+ (B) and Dnd1+/KO (C). These results suggest that testes in Dnd1+/KO males do not show significant germ cell deficiency. [file 1471-2156-14-54-S1.jpeg]

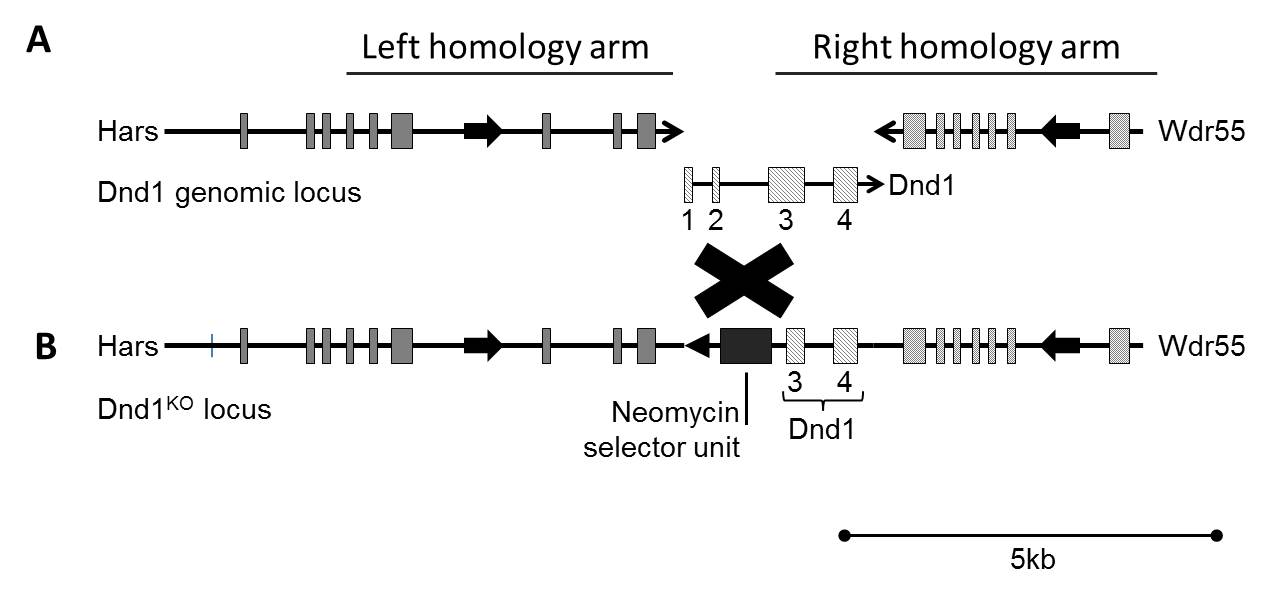

Supplement: Additional file 2: Figure S2 — Generation of Dnd1KO knockout mice. (A) Genomic Dnd1 locus showing flanking Hars and Wdr55 genes. Exons are shown as boxes and arrows show the direction of transcription. The left and right homology arms of the targeting construct are indicated. Following homologous recombination, exons 1, 2 and a portion of 3 of Dnd1 are removed and a neomycin selection unit is introduced. The triangle next to the neomycin unit indicates direction of transcription. The Hars and Wdr55 genes remain intact. [file 1471-2156-14-54-S2.jpeg]
